# Substance Use Screening, Brief Intervention, and Referral to Treatment Among Youth-Serving Clinicians

**DOI:** 10.1001/jamanetworkopen.2025.11579

**Published:** 2025-05-20

**Authors:** Kathleen Ragan-Burnett, Lyna Schieber, Andrew Terranella, Christina Mikosz

**Affiliations:** 1 Division of Overdose Prevention, National Center for Injury Prevention and Control, Centers for Disease Control and Prevention, Atlanta, Georgia

## Abstract

**Importance:**

Despite decreasing substance use by adolescents in recent years, overdose rates continue to rise. Morbidity and mortality from substance use is preventable if detected; however, screening practices among youth-serving clinicians are unknown.

**Objective:**

To describe youth-serving clinicians’ screening, brief intervention, and referral to treatment practices for substance use disorders (SUDs) among adolescents.

**Design, Setting, and Participants:**

This cross-sectional study used DocStyles data from September 5 to October 12, 2023, on clinical practice settings in the US. Responding clinicians included family physicians, internal medicine physicians, pediatricians, nurse practitioners, and physician assistants.

**Exposure:**

Delivery of services to youths aged 17 years or younger.

**Main Outcomes and Measures:**

Screening, brief intervention, and referral to treatment practices, including screening frequency and type of screening tool used, and screening at every well visit using a screening tool were assessed using descriptive statistics and multivariable logistic regression.

**Results:**

Of 1047 youth-serving clinicians (mean [SD] age, 45.3 [11.4] years; 555 male [53.0%]), 467 (44.6%) were family physicians, 250 (23.9%) were pediatricians, 132 (12.6%) were internal medicine physicians, 107 (10.2%) were nurse practitioners, and 91 (8.7%) were physician assistants (survey response rate, 57%). Median years in practice was 13 (IQR, 7-23 years). Most clinicians (634 [60.6%]) reported that skills in SUD diagnosis were relevant to their practice, and 800 (76.4%) reported seeing at least 1 adolescent with an SUD monthly. A majority of clinicians (596 [56.9%]) reported screening for SUDs at every well visit, with a high proportion of pediatricians reporting screening at every well visit (173 of 250 [69.2%]). Clinicians who screened at every well visit were more likely to use a screening tool (odds ratio, 1.87 [95% CI, 1.44-2.44]). Only 411 clinicians (39.3%) reported screening at every well visit using a screening tool; 321 clinicians (30.7%) offered all components of screening, brief intervention, and referral to treatment practice. Clinicians who reported seeing 5 or more adolescents with an SUD per month had a higher odds of screening with a standardized tool at every annual well visit (adjusted odds ratio, 2.19 [95% CI, 1.30-3.71]).

**Conclusions and Relevance:**

These findings suggest that while most clinicians report screening youths for SUDs at least sometimes, a substantial proportion screened only intermittently. Efforts to improve screening rates through education and systems-based practice changes may facilitate offering anticipatory guidance and SUD treatment in all youth-serving clinical settings.

## Key Points

**Question:**

What are the current practices surrounding substance use disorder screening, brief intervention, and referral to treatment practices among youth-serving clinicians in the US?

**Findings:**

In this cross-sectional study of 1047 youth-serving clinicians from multiple primary care specialties, 57% reported that they routinely screen adolescents for substance use disorders at every well visit. Only 39% reported using a screening tool at every well visit.

**Meaning:**

These findings suggest that substance use disorder screening in youths needs improvement to better adhere to recommended screening practices.

## Teaser

This cross-sectional study used survey data of clinicians from multiple primary care specialties to evaluate screening, brief intervention, and referral to treatment practices for substance use disorder among adolescents.

## Introduction

Substance use among adolescents is prevalent, with 5.3 million aged 12 to 17 years reporting a lifetime use of illicit drugs and 2.2 million reporting having a substance use disorder (SUD) in 2023.^1^ Adolescents who use drugs or who have an SUD are at higher risk for fatal and nonfatal overdose.^2^ Overdose deaths in adolescents aged 14 to 18 years rose 133% from 2019 to 2021,^3^ largely driven by synthetic opioids, including illegally made fentanyl.^3,4^ Nonfatal overdoses among adolescents have also risen.^5^ Substance use in adolescence is associated with early initiation of risky sexual behaviors, increased risk of sexually transmitted infections, and interpersonal violence.^6^

Morbidity and mortality associated with substance use among adolescents is preventable, and identifying adolescents who use substances and offering treatment or referral to care are important parts of primary care.^7^ Studies have documented success of screening and brief intervention programs among adolescents in reducing long-term substance use, SUD development, and health care use.^8,9^ The screening, brief intervention, and referral to treatment (SBIRT) model may be adapted and implemented in various primary care settings.^10,11^ As such, use of a screening tool for substance use and SUDs at every well visit or every visit regardless of purpose is recommended for adolescents by organizations such as the American Academy of Pediatrics^10^ and the Substance Abuse and Mental Health Services Administration (SAMHSA).^12^

Current screening practices among youth-serving clinicians are unknown. Previous studies from 1997 to 2014 reported screening rates from less than 50% to 86%.^13,14,15,16^ A recent survey of 471 primary care pediatricians found screening rates as high as 60%, with 42% using standardized tools.^17^ No recent study has reported on screening practices among youth-serving clinicians from multiple specialties. The objective of this study was to describe screening practices for SUDs and opioid use disorders (OUDs) among youth-serving clinicians in the US.

## Methods

### Study Population

This cross-sectional study was approved by the Centers for Disease Control and Prevention. Survey participants were provided a privacy policy to obtain informed consent. The study was conducted according to applicable federal laws and Centers for Disease Control and Prevention policy and followed the Strengthening of Reporting Observational Studies in Epidemiology (STROBE) reporting guideline for cross-sectional studies.^18^

Clinicians were surveyed through DocStyles from September 5 to October 12, 2023. DocStyles is a cross-sectional, unweighted, web-based panel survey commissioned by Porter Novelli Public Services^19^; conducted by M3 Global Research^20^; and developed by Porter Novelli Public Services with technical guidance from federal public health agencies and other clients. The full survey contained 120 questions on clinicians’ attitudes and behaviors regarding various health issues. This study used a subset of those questions. Respondents consisted of a main sample of primary care physicians and additional samples of other specialties (which might include non–primary care clinicians as Porter Novelli did not collect detailed specialty data). Eligible clinicians included those who practiced in the US; actively saw patients; worked in an individual, group, or hospital practice; and had been practicing for at least 3 years.

A total of 3108 health professionals were invited to participate to reach target quotas of 1000 primary care physicians (family physicians and internal medicine physicians), 250 obstetricians/gynecologists, 250 pediatricians, and 250 nurse practitioners or physician assistants. Of this sample, 1772 completed the survey, 313 did not complete the survey, 375 were excluded based on the screener questions or filled quotas, and 648 did not respond to the invitation or tried to respond after the survey closed. M3 Global Research’s panelists were verified using a double opt-in signup process with telephone confirmation at their place of work and invited via a link to the voluntary web-based survey. M3 Global Research sampled its currently active panel members on the basis of their activity level so that high responders (who answer >68% of surveys they are sent) were invited first, followed by medium responders (who answer 33%-67% of surveys sent) and low responders (who answer <33% of surveys sent). Respondents could exit the survey at any time and were paid an honorarium of $30 to $60 based on the number of questions they were asked to complete. Individual identifiers were not included to protect confidentiality. The overall response rate was 57%; pediatricians had the highest response rate (69%), followed by physician assistants (61%), nurse practitioners (57%), and family physicians and internal medicine physicians (53%). Given this study’s focus on youth-serving clinicians, questions were not fielded to obstetricians/gynecologists.

### Measures

Youth-serving clinicians were defined as those who responded yes to the screening question, “Do you ever see pediatric patients (children aged 17 or younger)?” Race and ethnicity data were self-selected to provide a demographic description of clinicians; response options included American Indian or Alaska Native, Asian, Black or African American, Hispanic or Latino, Native Hawaiian or Pacific Islander, White, multiracial, and other. Clinicians were asked a series of 19 questions with prompts about delivering care to adolescents.

Screening frequency was assessed by asking, “When do you screen for substance use and SUDs in adolescents,” with multiple response options, including (1) at an initial visit, (2) at every well visit, (3) at intermittent well visits, (4) if there are concerns about risky behaviors, (5) when a parent/guardian raises concerns, (6) at every visit regardless of purpose, and (7) never screen for SUDs in adolescents. Use of a screening tool to screen for SUDs in adolescents was defined as endorsing use of at least 1 of the following: (1) Screening to Brief Intervention^21^; (2) Brief Screener for Tobacco, Alcohol, and Other Drugs^22^; (3) Car, Relax, Alone, Forget, Friends, Trouble (CRAFFT) questionnaire^23^; (4) Tobacco, Alcohol, Prescription Medication, and Other Substance Use Tool (TAPS)^24^; (5) a tool developed by the clinician’s practice; or (6) a tool not mentioned. Concordance with recommended screening practices was defined as screening at every well visit or every visit regardless of purpose using a screening tool per American Academy of Pediatrics and SAMHSA guidelines.

### Statistical Analysis

We generated percentages for demographic, practice, and substance use screening characteristics among clinicians overall and by specialty. Statistical differences related to clinician characteristics, screening frequency, and behaviors were assessed using χ^2^ tests. Multivariable logistic regression was used to examine associations of clinician characteristics with reporting concordance with recommended screening practices for SUDs in adolescents, adjusting for demographic and clinical characteristics. Variance inflation factors were used to check multicollinearity of the variables in the multivariable logistic regression model. No multicollinearity was detected.^25^ Hypothesis testing was 2-tailed, with statistical significance set at *P* < .05. Data were analyzed using SAS, version 9.4 (SAS Institute Inc).

## Results

### Demographics

A total of 1047 youth-serving clinicians responded (mean [SD] age, 45.3 [11.4] years; 479 female [45.8%] and 555 male [53.0%]), including 467 family physicians (44.6%), 132 internal medicine physicians (12.6%), 250 pediatricians (23.9%), 107 nurse practitioners (10.2%), and 91 physician assistants (8.7%). Most clinicians were not Hispanic or Latino (982 [93.8%] compared with 65 of Hispanic or Latino ethnicity [6.2%]), White race (731 [69.8%] compared with 4 of American Indian or Alaska Native [0.4%], 198 Asian [18.9%], 42 Black or African American [4.0%], 4 Native Hawaiian or Pacific Islander [0.4%], 45 multiracial [4.3%], and 23 other [2.2%] race), younger than 50 years (677 [64.8%]), mainly worked in a group outpatient practice (681 [65.0%]), and practiced in suburban (526 [50.2%]) or urban (386 [36.9%]) areas. The median years of practice was 13 (IQR, 7-23) years, with the highest proportion practicing 20 years or longer being family physicians (202 of 467 [43.3%]) and the lowest being physician assistants (13 of 91 [14.3%]). Clinicians’ sex, age, race and ethnicity, main work setting, years of practice, region, and metropolitan area were significantly different across specialties (Table).

**Table.** Characteristics of Responding Youth-Serving Clinicians, US, 2023^a^

Characteristic	Clinicians, No. (%)						*P* value
	Total	Family physician	Internist	Pediatrician	Nurse practitioner	Physician assistant	
Total	1047 (100)	467 (44.6)	132 (12.6)	250 (23.9)	107 (10.2)	91 (8.7)	NA
Sex^b^							
Female	479 (45.8)	158 (33.8)	41 (31.1)	125 (50.0)	90 (84.1)	65 (71.4)	<.001
Male	555 (53.0)	304 (65.1)	88 (66.7)	121 (48.4)	16 (15.0)	26 (28.6)	
Age, mean (SD), y	45.3 (11.4)	47.9 (11.7)	46.3 (12.0)	44.6 (10.8)	40.9 (8.6)	38.0 (8.4)	NA
Age group, y							
23-29	33 (3.2)	4 (0.9)	4 (3.0)	6 (2.4)	8 (7.5)	11 (12.1)	<.001
30-39	389 (37.2)	145 (31.1)	45 (34.1)	104 (41.6)	44 (41.1)	51 (56.0)	
40-49	255 (24.4)	109 (23.3)	29 (22.0)	59 (23.6)	39 (36.5)	19 (20.9)	
50-59	222 (21.2)	117 (25.1)	29 (22.0)	54 (21.6)	14 (13.1)	8 (8.8)	
≥60	148 (14.1)	92 (19.7)	25 (18.9)	27 (10.8)	2 (1.9)	2 (2.2)	
Ethnicity							
Hispanic or Latino	65 (6.2)	32 (6.9)	8 (6.1)	18 (7.2)	4 (3.7)	3 (3.3)	.52
Not Hispanic or Latino	982 (93.8)	435 (93.2)	124 (93.9)	232 (92.8)	103 (96.3)	88 (96.7)	
Race^c^							
American Indian or Alaska Native	4 (0.4)	3 (0.6)	0	0	1 (0.9)	0	<.001
Asian	198 (18.9)	104 (22.3)	38 (28.8)	44 (17.6)	3 (2.8)	9 (9.9)	
Black or African American	42 (4.0)	17 (3.6)	3 (2.3)	10 (4.0)	8 (7.5)	4 (4.4)	
Native Hawaiian or Pacific Islander	4 (0.4)	1 (0.2)	1 (0.8)	2 (0.8)	0	0	
White	731 (69.8)	317 (67.9)	75 (56.8)	179 (71.6)	88 (82.2)	72 (79.1)	
Multiracial	45 (4.3)	16 (3.4)	11 (8.3)	8 (3.2)	6 (5.6)	4 (4.4)	
Other	23 (2.2)	9 (1.9)	4 (3.0)	7 (2.8)	1 (0.9)	2 (2.2)	
Main work setting							
Group outpatient practice	681 (65.0)	355 (76.0)	55 (41.7)	162 (64.8)	47 (43.9)	62 (68.1)	<.001
Individual outpatient practice	211 (20.2)	98 (21.0)	42 (31.8)	28 (11.2)	25 (23.4)	18 (19.8)	
Inpatient practice	155 (14.8)	14 (3.0)	35 (26.5)	60 (24.0)	35 (32.7)	11 (12.1)	
Years of practice							
Median (IQR)	13 (7-23)	16 (8-25)	16.15 (7-23)	12 (7-23)	11 (6-15)	9 (5-14)	NA
Range							
3-4	95 (9.1)	35 (7.5)	12 (9.1)	18 (7.2)	13 (12.2)	17 (18.7)	<.001
5-8	243 (23.2)	94 (20.1)	29 (22.0)	67 (26.8)	30 (28.0)	23 (25.3)	
9-14	227 (21.7)	87 (18.6)	25 (18.9)	54 (21.6)	30 (28.0)	31 (34.1)	
15-19	117 (11.2)	49 (10.5)	15 (11.4)	30 (12.0)	16 (15.0)	7 (7.7)	
≥20	365 (34.9)	202 (43.3)	51 (38.6)	81 (32.4)	18 (16.8)	13 (14.3)	
Region							
South	353 (33.7)	144 (30.8)	45 (34.1)	80 (32.0)	46 (43.0)	38 (41.8)	.001
Midwest	253 (24.2)	136 (29.1)	26 (19.7)	52 (20.8)	23 (21.5)	16 (17.6)	
Northeast	226 (21.6)	84 (18.0)	38 (28.8)	63 (25.2)	28 (26.2)	13 (14.3)	
West	215 (20.5)	103 (22.1)	23 (17.4)	55 (22.0)	10 (9.4)	24 (26.4)	
Metropolitan area							
Suburban	526 (50.2)	251 (53.8)	60 (45.5)	124 (49.6)	42 (39.3)	14 (15.4)	<.001
Urban	386 (36.9)	138 (29.6)	61 (46.2)	107 (42.8)	52 (48.6)	28 (30.8)	
Rural	135 (12.9)	78 (16.7)	11 (8.3)	19 (7.6)	13 (12.2)	14 (15.4)	
Relevance of skills in diagnosis and treatment of SUDs							
Extremely relevant or relevant	634 (60.6)	293 (62.7)	88 (66.7)	141 (56.4)	72 (67.3)	40 (44.0)	<.001
Somewhat relevant	291 (27.8)	136 (29.1)	28 (21.2)	80 (32.0)	17 (15.9)	30 (33.0)	
Not relevant at all	122 (11.7)	38 (8.1)	16 (12.1)	29 (11.6)	18 (16.8)	21 (23.1)	
No. of patients with an SUD per mo							
0	215 (20.5)	95 (20.3)	27 (20.5)	44 (17.6)	19 (17.8)	30 (33.0)	<.001
1-5	446 (42.6)	201 (43.0)	42 (31.8)	137 (54.8)	33 (30.8)	33 (36.3)	
≥5	354 (33.8)	159 (34.1)	61 (46.2)	63 (25.2)	48 (44.9)	23 (25.3)	
Do not know	32 (3.1)	12 (2.6)	2 (1.5)	6 (2.4)	7 (6.5)	5 (5.5)	
No. of patients with OUD per mo							
0	500 (47.8)	223 (47.8)	45 (34.1)	153 (61.2)	35 (32.7)	44 (48.4)	<.001
1-5	325 (31.0)	146 (31.3)	43 (32.6)	74 (29.6)	35 (32.7)	27 (29.7)	
≥5	188 (18.0)	86 (18.4)	42 (31.8)	14 (5.6)	32 (29.9)	14 (15.4)	
Do not know	34 (3.3)	12 (2.6)	2 (1.5)	9 (3.6)	5 (4.7)	6 (6.6)	

Abbreviations: NA, not applicable; OUD, opioid use disorder; SUD, substance use disorder.

^a^ Data were calculated based on DocStyles clinician data from September 5 to October 12, 2023.

^b^ Data do not add to 100% due to an unreported third response option.

^c^ The multiracial and other race categories are not grouped and represent the original survey response options.

### Practice Relevance

Overall, most clinicians (634 [60.6%]) reported that skills in the diagnosis and treatment of SUDs in adolescents in their practice were extremely relevant or relevant (Table); however, perception of relevance of skills significantly differed across specialties. For example, most physician assistants reported that skills were somewhat relevant (30 [33.0%]) or not relevant at all (21 [23.1%]) (*P* < .001). When asked about the average number of adolescents with an SUD seen per month, 800 clinicians (76.4%) reported seeing at least 1, with significantly higher proportions of internal medicine physicians (61 of 132 [46.2%]) and nurse practitioners (48 of 107 [44.9%]) reporting seeing 5 or more adolescents with an SUD per month (*P* < .001). Almost one-half of clinicians overall (513 [49.0%]) reported seeing at least 1 adolescent with an OUD per month, with significant differences across specialties. Most pediatricians (153 of 250 [61.2%]) reported seeing no adolescents with OUD, and significantly higher proportions of internal medicine physicians (42 [31.8%]) and nurse practitioners (32 [29.9%]) reported 5 or more adolescents with OUD than other specialties (*P* < .001).

### Screening Implementation and SBIRT

**Figure 1.**
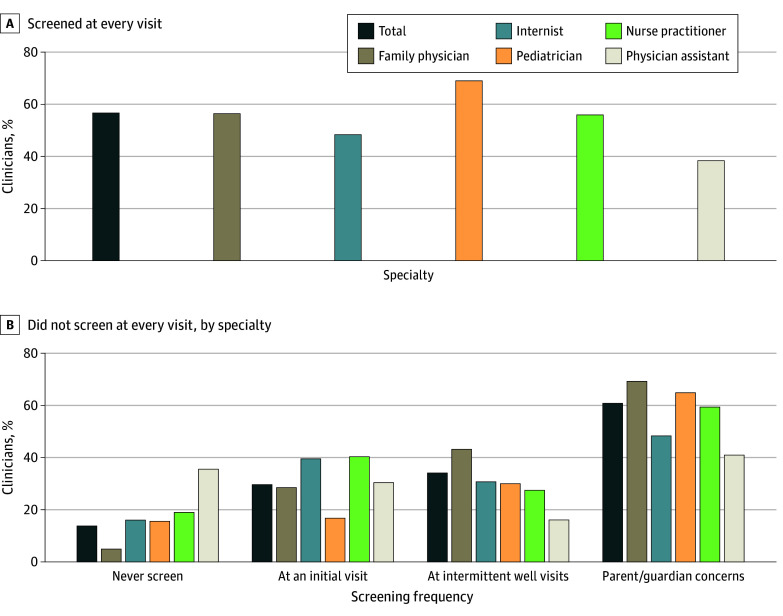
Screening for Substance Use and Substance Use Disorders in Adolescents by Specialty Data were calculated based on DocStyles clinician data from September 5 to October 12, 2023. eTable 1 in Supplement 1 provides detailed data on proportions by specialty.

A total of 596 clinicians (56.9%) reported screening at every well visit, and a few (88 [8.4%]) reported screening at every visit regardless of purpose (Figure 1A; eTable 1 in Supplement 1). A higher proportion of pediatricians endorsed screening at every well visit compared with other specialties (173 of 250 [69.2%]), and a higher proportion of nurse practitioners endorsed screening at every visit regardless of purpose compared with other specialties (26 of 107 [43.3%]). Among clinicians who reported not screening at every well visit (451 [43.1%]), 134 (29.7%) endorsed screening at an initial visit, 154 (34.2%) endorsed screening at intermittent well visits, 275 (61.0%) endorsed screening if there were concerns about risky behaviors or when a parent/guardian raised concerns, and 62 (13.8%) endorsed never screening for an SUD in adolescents (Figure 1B; eTable 1 in Supplement 1). Most physician assistants (56 of 91 [61.5%]) reported not screening at every well visit; of those, 20 (35.7%) reported never screening for SUDs.

**Figure 2.**
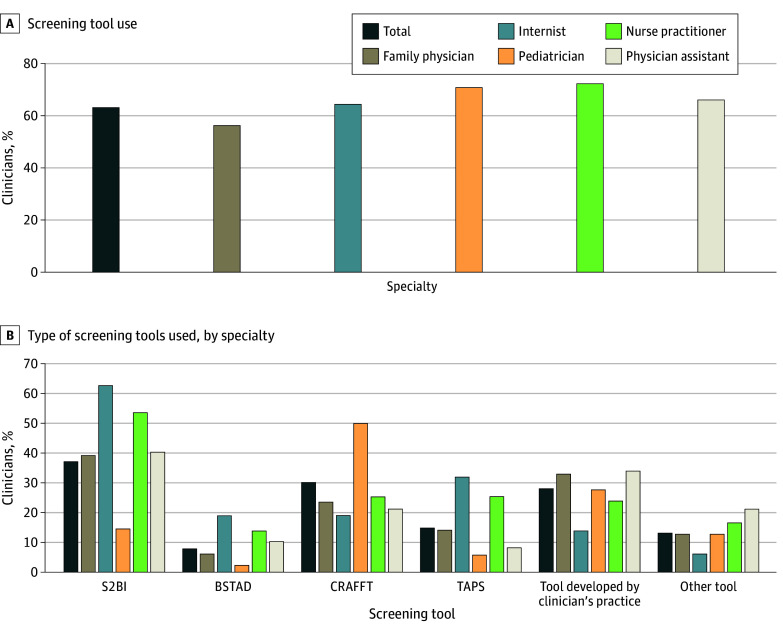
Screening Tool Use for Substance Use Disorders in Adolescents by Specialty Data were calculated based on DocStyles clinician data from September 5 to October 12, 2023. eTable 1 in Supplement 1 provides detailed data on proportions by specialty. BSTAD indicates Brief Screener for Tobacco, Alcohol, and Other Drugs^22^; CRAFFT, Car, Relax, Alone, Forget, Friends, Trouble^23^; S2BI, Screening to Brief Intervention^21^; TAPS, Tobacco, Alcohol, Prescription Medication, and Other Substance Use.^24^

A total of 363 clinicians (36.9%) who screened for an SUD reported not using a screening tool (Figure 2A; eTable 1 in Supplement 1). Among clinicians who used a screening tool (622 [63.2%]), the most endorsed tools were Screening to Brief Intervention (232 [37.3%]) and CRAFFT (189 [30.4%]) (Figure 2B; eTable 1 in Supplement 1). Types of screening tools used varied by specialty. Internal medicine physicians were more likely to use the Screening to Brief Intervention (49 of 78 [62.8%]) and TAPS (25 of 78 [32.1%]), 85 of 169 pediatricians (50.3%) used CRAFFT, and 85 of 257 family physicians (33.1%) and 16 of 47 physician assistants (34.0%) reported using a tool developed by their practice.

**Figure 3.**
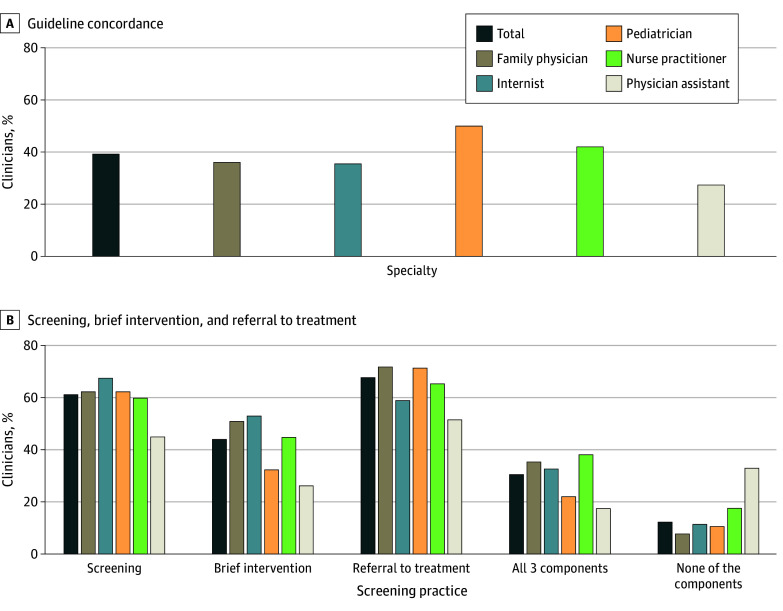
Concordance With Recommended Screening Practices by Specialty Data were calculated based on DocStyles clinician data from September 5 to October 12, 2023. eTable 1 in Supplement 1 provides detailed data on proportions by specialty.

Clinicians who reported use of a screening tool had greater odds of screening at every well visit (odds ratio [OR], 1.87 [95% CI, 1.44-2.44]) (eTable 2 in Supplement 1). Overall, 411 clinicians (39.3%) reported screening at every well visit and using a screening tool (Figure 3A). Pediatricians had the highest proportion of respondents (125 of 250 [50.0%]) who followed recommended screening practices, and physician assistants had the lowest (25 of 91 [27.5%]).

When asked about treating adolescents with SUDs using the SBIRT model, 641 clinicians (61.2%) reported providing screening, 461 (44.0%) reported providing brief intervention, 710 (67.8%) reported providing referral to treatment, and 321 (30.7%) endorsed all 3 services (Figure 3B; eTable 1 in Supplement 1). Compared with 128 clinicians overall (12.2%), 30 of 91 physician assistants (33.0%) reported not providing any SBIRT services. Most clinicians (612 [58.1%]) reported that their practice required some sort of consent to provide screening for patients younger than 18 years for OUD (eTable 1 in Supplement 1).

### Regression Modeling

**Figure 4.**
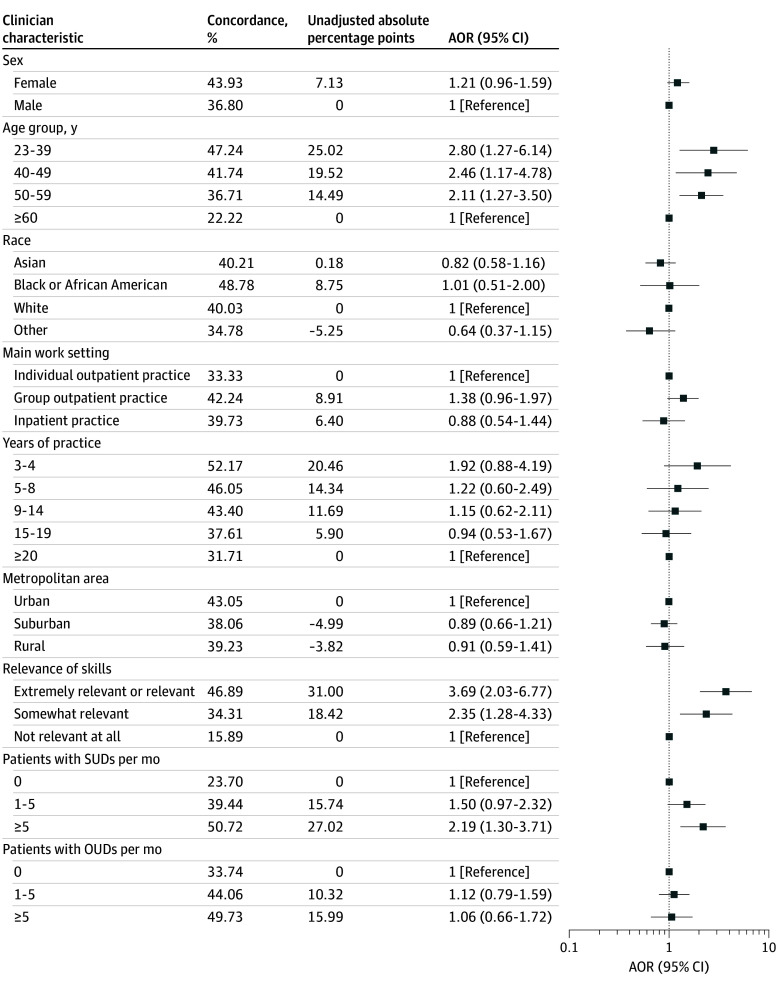
Associations of Youth-Serving Clinicians’ Characteristics With Reporting Concordance With Recommended Screening Practices (N = 1047) The adjusted odds ratios (AORs) were calculated using a multivariable logistic regression model adjusted for the demographic and clinical characteristics shown. The unadjusted absolute difference is the difference between the percentage of each category reporting concordance with recommended screening practices and the percentage of the reference category. OUD indicates opioid use disorder; SUD, substance use disorder.

Compared with clinicians aged 60 years or older, younger clinicians had significantly higher odds of reporting recommended screening practices (aged 23 to 39 years: adjusted OR [AOR], 2.80 [95% CI, 1.27-6.14]; aged 40-49 years: AOR, 2.46 [95% CI, 1.17-4.78]; aged 50-59 years: AOR, 2.11 [95% CI, 1.27-3.50]) (Figure 4; eTable 3 in Supplement 1). Compared with clinicians who reported that skills in diagnosis and treatment of adolescents with SUDs were not relevant at all, those who reported that relevance of skills was either extremely relevant or relevant had a significantly higher odds of reporting recommended screening practices (AOR, 3.69 [95% CI, 2.03-6.77]). Compared with clinicians who reported seeing no adolescents with an SUD per month, those who reported seeing 5 or more had a significantly higher odds of reporting recommended screening practices (AOR, 2.19 [95% CI, 1.30-3.71]).

## Discussion

This cross-sectional study reports survey results on substance use SBIRT practices among youth-serving clinicians of multiple specialties. While most clinicians (56.9%) screened adolescents at every well visit, the majority of youth-serving clinicians (61.0%) were not following recommended screening practices endorsed by SAMHSA and the American Academy of Pediatrics, including screening at well visits using a screening tool, and most did not use SBIRT routinely.

Though substance use overall has declined in recent years,^26^ overdoses among adolescents have risen.^3^ Physician awareness of substance use behaviors among adolescent patients is essential to offering appropriate substance use education, treatment, and anticipatory guidance on overdose prevention.^27^ Consistent with the import of substance use in pediatric practice, most clinicians reported treating at least 1 adolescent with an SUD per month, and most were screening for SUDs at least sometimes. Most clinicians were also implementing at least some components of SBIRT, and a majority of clinicians reported that skills in diagnosis and treatment of SUDs were relevant to their practice.

While these results are promising, there is room for improvement. Among all clinicians, 43.1% reported not screening at every well visit. Among those who reported any screening behaviors, 36.9% did not use a screening tool. Only 39.3% of clinicians reported screening using a tool at all well visits. Thirty-four percent of clinicians only screened for SUD intermittently, and many of these clinicians may have been assessing patients by clinical impression alone. However, clinical impressions frequently underestimate both presence and severity of SUDs in adolescents.^28^

Our results are consistent with a recent survey of 471 board-certified pediatricians that found that 60% of these physicians always screened adolescents during annual well visits and 42% used a standardized tool,^17^ which is an improvement over a study that used 2014 survey data among pediatricians and found that only 26% screened using structured tools.^16^ Our study adds to the body of literature by including youth-serving clinicians from multiple disciplines. The findings show wide variation in screening practices by specialty and perception of relevance, with a higher proportion of pediatricians than other disciplines following best practice recommendations. This variation may stem from conflicting screening recommendations. While SAMHSA and the American Academy of Pediatrics recommend routine SUD screening in adolescents,^10,12^ the US Preventive Services Task Force and American Academy of Family Physicians have concluded that evidence is insufficient to support routine substance use screening in adolescents.^29^ Consent requirements for screening also varied by specialty, with more pediatricians reporting that no specific consent was required for screening compared with other specialties, and may also account for screening variation by specialty. Adolescent confidentiality is an important component of pediatric training, and several organizations support confidential screening for substance use among adolescents.^10,30^ Ensuring that all youth-serving clinicians are trained in confidential care may improve screening rates across specialties.^31^

It has been speculated that many, if not most, adolescents who use substances and who have SUDs remain undiagnosed.^1,32^ Screening is an important component of a cascade of care to identify patients at risk for SUDs and in need of treatment, particularly treatment for OUD.^33,34,35^ In 2023, of the 2.2 million adolescents aged 12 to 17 years with a past-year SUD, only one-half (1.1 million) reported receiving any treatment.^36^ In 2023, among adolescents with an OUD, only 7.5% received medication treatment.^36^ Analyses of pharmacy and claims data have found similarly low treatment rates and dispensing of buprenorphine for OUD treatment.^32,37,38,39^ In addition to increasing diagnoses of SUDs, more screening might increase opportunities to offer interventions to reduce adverse outcomes, such as making naloxone available to all youth at risk of overdose. Considering the prevalence of counterfeit pills,^40^ youths who use substances may benefit from low-barrier access to naloxone, including via a prescription. Finally, screening may normalize the discussion of substance use from an early age, which may build trust and facilitate later educational, treatment, and adverse outcomes discussions between adolescents and clinicians.

Clinicians who reported using a screening tool were more likely to screen at all well visits, and efforts to make screening tools more readily available and easier to use in primary care clinics may lead to increased use. Multiple studies have shown the feasibility and effectiveness of systems-driven changes, such as integration of evidence-based screening tools, into the electronic health record to increase screening rates.^41,42^

Systems-based approaches to improve adolescent substance use screening rates across all specialties, as well as to improve SBIRT model implementation, may substantially improve substance use outcomes for adolescents. Studies of adolescents treated in primary care clinics using SBIRT models have found lower odds of any substance use and lower health care use that persisted into adulthood.^9^ However, there are challenges to implementation of SBIRT models. In our study, less than one-half of clinicians offered brief interventions, with 32.2% reporting not referring patients for treatment and only 30.7% reporting using all SBIRT components with adolescents. A recent study of SBIRT implementation sites (including 392 primary care sites) found SBIRT to be poorly implemented, with only 5% offering brief interventions and 1% offering referrals for treatment when indicated.^43^

Improving clinician education on adolescent substance use and screening across all youth-serving specialties may also increase screening rates. A study of 120 pediatric residency programs found that while most programs (83%) offered some education on adolescent substance use, less than one-half (41%) included SBIRT training.^44^ Another study that looked more broadly at knowledge and comfort in delivering confidential care for adolescents among family medicine residents found that 50% reported no formal training in residency and that formal training was associated with a higher likelihood of always delivering confidential adolescent care.^31^ Additional studies of medical residents and community clinicians have shown improvements in screening practices through the addition of an SUD curriculum during residency,^45^ asynchronous trainings,^46^ and short continuing medical education sessions.^47^

### Limitations

This study has several limitations. First, due to survey methodology (eg, clinicians with a greater likelihood of responding were contacted first) and small sample sizes among specialties, respondents may not be nationally representative of all family physicians, internal medicine physicians, pediatricians, nurse practitioners, and physician assistants, thus limiting the generalizability of the findings. Second, Porter Novelli Public Services does not collect details on the specialty of nurse practitioners or the practice settings of physician assistants. Approximately 70% of nurse practitioners and 22% of physician assistants in the US deliver primary care.^48,49^ Third, due to survey constraints, there was limited ability to obtain details about screening behaviors, such as motivations for or barriers to specific practices. In addition, it is unknown whether screening tools developed by a clinician’s practice or those endorsed as other were validated. Fourth, there was no ability to assess temporality or directionality, which may inhibit a thorough understanding of the associations among screening behaviors, screening tool use, and burden of patients with SUDs or OUDs.

## Conclusions

In this cross-sectional study, the findings suggest that screening for SUDs and implementation of SBIRT models among youth-serving clinicians could be improved. Identifying youths with SUDs and referring them to treatment remain crucial components to reduce substance use–related morbidity and mortality, including overdose. Systems-related interventions, such as adoption of evidence-based screening tools integrated in the electronic health record, may improve screening rates. Screening offers opportunities for clinicians to engage in primary prevention of SUDs through anticipatory guidance, refer patients who use substances to appropriate care, and offer interventions to reduce adverse outcomes to all at-risk youth.

## Notes

Supplement 1. **eTable 1.** Substance Use Screening in Adolescents by Clinician Specialty, US, 2022 **eTable 2.** Associations Between Screening Tool Use and Screening for Substance Use and Substance Use Disorders at Every Well Visit **eTable 3.** Multivariable Logistic Regression Model Examining Associations of Youth-Serving Clinicians’ Characteristics With Reporting Concordance With Recommended Screening Practices

Supplement 2.
Data Sharing Statement
