# Supplementary material for: Substance Use Screening, Brief Intervention, and Referral to Treatment Among Youth-Serving Clinicians
Source: JAMA Netw Open. 2025 May 20;8(5):e2511579. doi: 10.1001/jamanetworkopen.2025.11579 (PMC12093189; doi:10.1001/jamanetworkopen.2025.11579)
Supplement: Supplement 1. — eTable 1. Substance Use Screening in Adolescents by Clinician Specialty, US, 2022 eTable 2. Associations Between Screening Tool Use and Screening for Substance Use and Substance Use Disorders at Every Well Visit eTable 3. Multivariable Logistic Regression Model Examining Associations of Youth-Serving Clinicians’ Characteristics With Reporting Concordance With Recommended Screening Practices [file jamanetwopen-e2511579-s001.pdf]

Supplemental Online Content

Ragan-Burnett K, Schieber L, Terranella A, Mikosz C. Substance use screening, brief intervention, and treatment referral among youth-serving clinicians. *JAMA Netw Open*. 2025;8(5);e2511579. doi:10.1001/jamanetworkopen.2025.11579

eTable 1. Substance Use Screening in Adolescents by Clinician Specialty, US, 2022

eTable 2. Associations Between Screening Tool Use and Screening for Substance Use and Substance Use Disorders at Every Well Visit

eTable 3. Multivariable Logistic Regression Model Examining Associations of Youth-Serving Clinicians' Characteristics With Reporting Concordance With Recommended Screening Practices

This supplemental material has been provided by the authors to give readers additional information about their work.

© 2025 Ragan-Burnett K et al. *JAMA Network Open*.

eTable 1. Substance Use Screening in Adolescents by Clinician Specialty, US, 2022

Clinician characteristic		Total	Family Practitioner	Internist	Pediatrician	Nurse Practitioner	Physician Assistant
**Total**		1047	467	132	250	107	91
**Screening for substance use and SUDs in adolescents**							
Screen at every well visit		596 (56.92)	264 (56.53)	64 (48.48)	173 (69.20)	60 (56.07)	35 (38.46)
At every visit regardless of purpose		88 (14.77)	25 (9.47)	10 (15.63)	18 (10.40)	26 (43.33)	9 (5.87)
Do not screen at every well visit		451 (43.08)	203 (43.47)	68 (51.52)	77 (30.80)	47 (43.93)	56 (61.54)
At an initial visit		134 (29.71)	58 (28.57)	27 (39.71)	13 (16.88)	19 (40.43)	17 (30.36)
At intermittent well visits		154 (34.15)	88 (43.35)	21 (30.88)	23 (29.87)	13 (27.66)	9 (16.07)
If there are concerns about risky behaviors or when a parent/guardian raises concerns		275 (60.98)	141 (69.46)	33 (48.53)	50 (64.94)	28 (59.57)	23 (41.07)
I never screen for SUD in adolescents		62 (13.75)	10 (4.93)	11 (16.18)	12 (15.58)	9 (19.15)	20 (35.71)
**Screening tool use to screen for SUDs in adolescents (n=985)**							
Yes[a]		622 (63.15)	257 (56.24)	78 (64.46)	169 (71.01)	71 (72.45)	47 (66.20)
Screening to Brief Intervention (S2BI)		232 (37.30)	101 (39.30)	49 (62.82)	25 (14.79)	38 (53.52)	19 (40.43)
Brief Screener for Tobacco, Alcohol, and other Drugs (BSTAD)		50 (8.04)	16 (6.23)	15 (19.23)	4 (2.37)	10 (14.08)	5 (10.64)
Car; Relax; Alone; Forget; Friends; Trouble (CRAFFT)		189 (30.39)	61 (23.74)	15 (19.23)	85 (50.30)	18 (25.35)	10 (21.28)
Tobacco, Alcohol, Prescription medication, and other Substance use tool (TAPS)		94 (15.11)	37 (14.40)	25 (32.05)	10 (5.92)	18 (25.35)	4 (8.51)
Other tool developed by clinician's practice		176 (28.30)	85 (33.07)	11 (14.10)	47 (27.81)	17 (23.94)	16 (34.04)
Other tool, name not mentioned		82 (13.18)	33 (12.84)	5 (6.41)	22 (13.02)	12 (16.90)	10 (21.28)
No [I do not use a screening tool]		363 (36.85)	200 (43.76)	43 (35.54)	69 (28.99)	27 (27.55)	24 (33.80)
**Concordance with recommended screening practices***							
Yes, screens at every well visit and uses a screening tool		411 (39.26)	169 (36.19)	47 (35.61)	125 (50.00)	45 (42.06)	25 (27.47)
No		636 (60.74)	298 (63.81)	85 (64.39)	125 (50.00)	62 (57.94)	66 (72.53)

© 2025 Ragan-Burnett K et al. *JAMA Network Open*.

eTable 1. Substance Use Screening in Adolescents by Clinician Specialty, US, 2022 (continued)

Clinician characteristic		Total	Family Practitioner	Internist	Pediatrician	Nurse Practitioner	Physician Assistant
**Total**		1047	467	132	250	107	91
**Screening, Brief Intervention, and Referral to Treatment (SBIRT)[a]**							
I provide screening		641 (61.22)	291 (62.31)	89 (67.42)	156 (62.40)	64 (59.81)	41 (45.05)
I provide brief intervention		461 (44.03)	238 (50.96)	70 (53.03)	81 (32.40)	48 (44.86)	24 (26.37)
I provide referral to treatment		710 (67.81)	336 (71.95)	78 (59.09)	179 (71.60)	70 (65.42)	47 (51.65)
I provide screening, brief intervention, and referral to treatment		321 (30.66)	165 (35.33)	43 (32.58)	56 (22.40)	41 (38.32)	16 (17.58)
I do not provide any of the above for SUD		128 (12.23)	37 (7.92)	15 (11.36)	27 (10.80)	19 (17.76)	30 (32.97)
**Consent required to provide screening for patients under 18 years old for OUD**							
Parental consent only required		137 (13.09)	72 (15.42)	16 (12.12)	22 (8.80)	15 (14.02)	12 (13.19)
Minor consent only required		118 (11.27)	42 (8.99)	18 (13.64)	48 (19.20)	4 (3.74)	6 (6.59)
Either parental or minor consent required		119 (11.37)	61 (13.06)	22 (16.67)	21 (8.40)	9 (8.41)	6 (6.59)
Both parental and minor consent required		238 (22.73)	120 (25.70)	30 (22.73)	26 (10.40)	41 (38.32)	21 (23.08)
No specific consent requirements		435 (41.55)	172 (36.83)	46 (34.85)	133 (53.20)	38 (35.51)	46 (50.55)

[a]Data do not add to 100% because respondents were instructed to select all response choices that apply.

Abbreviations: SUD, substance use disorder; OUD, opioid use disorder.

© 2025 Ragan-Burnett K et al. *JAMA Network Open*.

eTable 2. Associations Between Screening Tool Use and Screening for Substance Use and Substance Use Disorders at Every Well Visit

Screening tool use	Screening for substance use and SUDs at every well visit			
	Yes	No	OR (95% CI)	*P* value
Any tool[a]				
Yes	411 (68.96)	211 (54.24)	1.87 (1.44-2.44)	<.0001
No	185 (31.04)	178 (45.76)		
Common standardized tool[b]				
Yes	291 (48.83)	146 (32.37)	1.99 (1.55-2.57)	<.0001
No	305 (51.17)	305 (67.63)		

Abbreviations: SUDs, substance use disorders; OR, odds ratio; CI, confidence interval.

[a]Use of "any" screening tool to screen for SUDs in adolescents was defined as endorsing use of at least one of the following response options: 1) Screening to Brief Intervention (S2BI) 2) BSTAD (Brief Screener for Tobacco, Alcohol, and other Drugs) 3) CRAFFT (Car; Relax; Alone; Forget; Friends; Trouble) (4) TAPS (Tobacco, Alcohol, Prescription medication, and other Substance use Tool) 5) other tool developed by clinician's practice 6) other tool not mentioned.

[b]"Common standardized tool" included endorsing use of at least one of the following: 1) S2BI 2) BSTAD 3) CRAFFT 4) TAPS

© 2025 Ragan-Burnett K et al. *JAMA Network Open*.

eTable 3. Multivariable Logistic Regression Model Examining Associations of Youth-Serving Clinicians' Characteristics With Reporting Concordance With Recommended Screening Practices

Clinician characteristic	Total (n=1047)	
	Unadjusted OR (95% CI)	Adjusted OR (95% CI)
**Sex**		
Female	1.35 (1.04-1.74)	1.21 (0.96-1.59)
Male	Reference	Reference
**Age (years)**		
23 to 39	3.13 (2.02-4.86)	2.80 (1.27-6.14)
40 to 49	2.51 (1.66-4.01)	2.46 (1.17-4.78)
50 to 59	2.03 (1.25-3.29)	2.11 (1.27-3.50)
60 and older	Reference	Reference
**Race**		
White	Reference	Reference
Black or African American	1.43 (0.76-2.68)	1.01 (0.51-2.00)
Asian	1.01 (0.73-1.40)	0.82 (0.58-1.16)
Others	0.88 (0.48-1.34)	0.64 (0.37-1.15)
**Main work setting**		
Individual outpatient practice	Reference	Reference
Group outpatient practice	1.46 (1.05-2.04)	1.38 (0.96-1.97)
Inpatient practice	1.32 (0.85-2.05)	0.88 (0.54-1.44)
**Years of practice**		
3 to 4	2.23 (1.41-3.53)	1.92 (0.88-4.19)
5 to 8	1.76 (1.25-2.46)	1.22 (0.60-2.49)
9 to 14	1.60 (1.13-2.27)	1.15 (0.62-2.11)
15 to 19	1.32 (0.85-2.05)	0.94 (0.53-1.67)
20+	Reference	Reference
**Metro**		
Urban	Reference	Reference
Suburban	1.20 (0.80-1.79)	0.89 (0.66-1.21)
Rural	0.99 (0.67-1.47)	0.91 (0.59-1.41)
**Relevance of Skills in Diagnosis and Treatment of SUDs in Adolescents to your Practice**		
Extremely relevant or Relevant	4.90 (2.92-8.34)	3.69 (2.03-6.77)
Somewhat relevant	2.86 (1.64-5.0)	2.35 (1.28-4.33)
Not relevant at all	Reference	Reference
**# Patients with SUD Per Month**		
0	Reference	Reference
1-5	2.08 (1.44-3.01)	1.50 (0.97-2.32)
5+	3.33 (2.28-4.87)	2.19 (1.30-3.71)

© 2025 Ragan-Burnett K et al. *JAMA Network Open*.

eTable 3. Multivariable Logistic Regression Model Examining Associations of Youth-Serving Clinicians' Characteristics With Reporting Concordance With Recommended Screening Practices (continued)

Clinician characteristic	Total (n=1047)	
	Unadjusted OR (95% CI)	Adjusted OR (95% CI)
**# Patients with OUD Per Month**		
0	Reference	Reference
1-5	1.55 (1.16-2.07)	1.12 (0.79-1.59)
5+	1.95 (1.38-2.74)	1.06 (0.66-1.72)

Abbreviations: OR, odds ratio; CI, confidence interval; SUD, substance use disorder; OUD, opioid use disorder.

© 2025 Ragan-Burnett K et al. *JAMA Network Open*.
