# Supplementary material for: Substance Use Screening, Brief Intervention, and Referral to Treatment Among Youth-Serving Clinicians
Source: JAMA Netw Open. 2025 May 20;8(5):e2511579. doi: 10.1001/jamanetworkopen.2025.11579 (PMC12093189; doi:10.1001/jamanetworkopen.2025.11579)
Supplement: Supplement 2. — Data Sharing Statement [file jamanetwopen-e2511579-s002.pdf]

Data Sharing Statement

Ragan-Burnett. Substance Use Screening, Brief Intervention, and Referral to Treatment Among Youth-Serving Clinicians. *JAMA Netw Open*. Published May 20, 2025. doi:10.1001/jamanetworkopen.2025.11579

Data
Data available: No
